# Supplementary material for: Fast alignment of mass spectra in large proteomics datasets, capturing dissimilarities arising from multiple complex modifications of peptides
Source: BMC Bioinformatics. 2023 Nov 8;24:421. doi: 10.1186/s12859-023-05555-y (PMC10631047; doi:10.1186/s12859-023-05555-y)

Supplementary data – Additional file 1 - Fast alignment of mass spectra in large proteomics datasets, capturing dissimilarities arising from multiple complex modifications of peptides

Grégoire Prunier[1,2], Mehdi Cherkaoui[1,2], Albane Lysiak[1,3], Olivier Langella[4], Melisande Blein-Nicolas[4], Virginie Lollier[1,2], Emile Benoist[3], Géraldine Jean[3], Guillaume Fertin[3], Hélène Rogniaux[1,2] and Dominique Tessier[1,2]

A. Extended description of parameters used to process the spectra dataset *DSim* by different software (Table 1) 1

B. In-depth evaluation of SpecGlobX with several additional simulated data sets 3

C. Extended description of parameters used to process the spectra dataset *HEK293* 3

D. Annotated Spectrum Plots 7

A. Extended description of parameters used to process the spectra dataset *DSim* by different software (Table 1)

A set of parameters are shared by the three software, even though they do not have the same name (SpecOMS, MSFragger and MODPlus)

The human proteome was downloaded from Ensembl 99, release GrCh38 on the Ensembl FTP server. Only proteins predicted with the annotation "protein coding" were kept. The contaminant proteins was downloaded from the cRAP contaminant database http://ftp.thegpm.org/fasta/cRAP/

```
databank= Homo_sapiens_GRCh38.fasta
contaminant= crapPeptidesRef.fasta

fragmentAccuracy=0.02 Da
enzyme = trypsin
fixed modification=57.021464@C
no decoy
minimumPeptideCharge=1
maximumPeptideCharge=3
1 missed cleavage allowed
peptide size between 7 and 30
```

Parameters specific to SpecOMS

The SpecOMS executable is available at https://github.com/dominique-tessier/SpecOMS

```
// The 60 most intense peaks are selected
maxMassesCount=60
// Only the best interpretation per spectrum is selected
single_match=true
// The comparison is between experimental spectra and
theoretical spectra
searchMode=projection
// Only the PSMs with at least 5 shared peaks are selected
before SpecFit
threshold=5
// When PSMs are ranked to interpret a spectrum, there is no
// bias towards ΔM=0
minimumScore=60
// Once a PSM is selected according to the number of shared
// peaks to interpret one spectrum, SpecFit tries to position
// ΔM on all sites in the peptide to generate different
// modified peptides in turn.
// PSM are re-ranked according to the new number of shared
// peaks
shift=true
// The lower bound of the precursor mass window
minMassDelta=-2000
```

Parameters specific to MODPlus

```
# 2 allows for arbitrary number of modifications per peptide.
NumMods=2
# Sets the minimum/maximum modification size in dalton to
consider. (Default values are -150,+350)
ModMassSize=-350,180
# ModAnnotation : 5 allows all Unimod modifications including
AA substitutions.
ModAnnotation=5
All other parameters were set to their default values.
```

Parameters specific to MSFragger

```
# Lower bound of the precursor mass window.
precursor_mass_lower = -150
# Upper bound of the precursor mass window
precursor_mass_upper = 500
# True precursor mass tolerance (window is +/- this value).
precursor_true_tolerance = 20 ppm
# Perform mass calibration (2 to find optimal parameters)
calibrate_mass = 2
# Do not use all variable modifications in first search
use_all_mods_in_first_search = 0
# Maximum total number of variable modifications per peptide.
max_variable_mods_per_peptide = 3
All other parameters were set to their default values.
```

B. In-depth evaluation of SpecGlobX with several additional simulated spectra datasets (Tables S1, S2, S3)

Table S1. Effect of missing peaks on *DSim1* and *DSim2* results

% Missing peaks	% PSM with neutral loss<br>*DSim1/DSim2*	%PSM with all modifications correct<br>*DSim1/DSim2*	% Correct modifications compared to expected<br>*DSim1/DSim2*
5	77/0	50/90	56/72
10	73/0	50/90	55/71
**20**	**68**/0	**50**/89	**53**/71
30	57/0	45/89	42/69
40	48/0	41/89	35/68
50	42/0	37/89	29/67

DSim1 and DSim2 datasets contain simulated spectra (50,000 spectra) generated from the same set of peptides with the same modifications as the *DSim* dataset (each occurrence of D and N are modified). However, each spectrum in all *DSim1* datasets has a neutral loss, while the spectra in all *DSim2* datasets have no neutral loss. Each column shows the percentage of hits relating to one computing criterion counted on the alignments returned by SpecGlobX for the *Dim1/DSim2* spectra datasets. Each row presents the results obtained by a variation of *DSim1* and *DSim2* spectra datasets obtained by changing the number of missing peaks removed randomly (2/3 b-ions and 1/3 y-ions) from 5% to 50%. Numbers in bold refer to the results reproduced from Table 1.

Not surprisingly, in Table S1 we can observe that SpecGlobX performance gradually decreases according to the percentage of missing peaks since the more peaks are missing, the more difficult it becomes to align without *a priori*. However, thanks to the completion mechanism, the degradation is slow when only D and N are modified, whereas it is fast when a neutral loss is added to each spectrum. Furthermore, due to the faster decline in correct modifications compared to the decline in correct PSMs, we can conclude that the performance degradation concerns the most modified peptides. Besides, we also note that the loss of peaks does not result in "not-aligned masses" (their number remains at 0, even with a 50% loss of peaks).

Table S2. Effect of amino acid fragmentation absence on *DSim1 and DSim2* results

# Missing aa fragmentations	% PSM with neutral loss	%PSM with all modifications correct	% Correct modifications compared to expected
1	77/0	50/89	55/72
2	71/0	52/83	52/64
3	64/0	49/78	46/57
4	56/0	46/73	40/50
5	49/0	42/68	33/42
6	42/0	37/62	28/35

DSim1 and DSim2 datasets contain simulated spectra (50,000 spectra) generated as explained in the Table S1 legend. Each column shows the percentage of hits relating to one computing criterion counted on the alignments returned by SpecGlobX for the *Dim1/DSim2* spectra datasets. Each row presents the results obtained by a variation of *DSim1* and *DSim2* spectra datasets obtained by changing the number of amino acid fragmentation missing peaks from 1 amino acid to 6 amino acids (both b-ion and y-ion referring to the same fragmentation are missing).

The approach we have implemented to compensate for the absence of fragmentation traces seems effective (when neither the b-ion nor the y-ion are in the spectrum), as SpecGlobX is resistant to a few missing fragmentation traces. However, as the number of fragmentation losses in the spectra increases, performance declines more rapidly compared with the loss of random peaks. Indeed, a loss of six amino acids represents only 25% to 50% of the total number of peaks generated by peptides ranging from 12 to 25 in length. Besides, it is also worth noting that the loss of fragmentation traces does not result in "non-aligned masses" (their number remains at 0, even with the loss of six amino acid traces).

Table S3. Effect of co-fragmentation on *DSim1/DSim2* results

CoFragmentation	% PSM with neutral loss<br>*DSim1/DSim2*	%PSM with all modifications correct<br>*DSim1/DSim2*	% Correct modifications compared to expected<br>*DSim1/DSim2*
NO	**68**/0	**50**/89	**53**/71
YES	55/0	35/83	39/44

In the first row, *DSim1* and *DSim2* datasets contain simulated spectra (50,000 spectra) generated as explained in the Table S1 legend. Numbers in bold refer to the results reproduced from Table 1 when 20% of random peaks are missing (2/3 b-ions, 1/3 y-ions). In the second row, a co-fragmented peptide without any modification is added to each spectrum. This co-fragmented peptide is obtained by randomly shuffling the peptide amino acid sequence except the last amino acid, which remains at the C-terminal side to preserve the peptide's tryptic properties. For example, the spectrum of the peptide SVAITISAIQIED[21.98]SAK is thus transformed by cumulating the fragmentation of the peptide IIVIAIDAESSSAQTK. If the total number of peaks after the addition of peaks is less than 60, random masses are added. Otherwise, excess peaks are randomly removed from the

shuffled peptide fragmentation. PSMs supplied to SpecGlobX refer only to the original peptide (SVAITISAIQIEDSAK in the previous example). Each column shows the percentage of hits relating to one computing criterion counted on the alignments returned by SpecGlobX.

Adding co-fragmentation to spectra degrades the results, in particular on peptides displaying many modifications. However, neutral losses are detected in more than half of the spectra, and more than a third of the shifts returned by SpecGlobX are correct, while all spectra contain simultaneously co-fragmented peptides, one to many modifications, and a loss of 20% of the peaks.

C. Extended description of parameters used to process the spectra dataset *HEK293*

We downloaded the 24 raw files from PRIDE (PXD001148), converted them in the mgf format using msConvert. The protein database was generated from the Human protein database GRCh37 from the Ensembl genome assembly. Next, we executed SpecOMS on those set of spectra with the following parameters:

databank= ***Homo_sapiens_GRCh37_61.fasta***

contaminant= ***crapPeptidesRef.fasta***

a decoy database is generated using reverse sequences

fragmentAccuracy=***0.02 Da***

enzyme = ***trypsin***

fixed modification=***57.021464@C***

minimumPeptideCharge=***1***

maximumPeptideCharge=***3***

1 missed cleavage allowed

// The 60 most intense peaks are selected
maxMassesCount=***60***

// Only the best interpretation per spectrum is selected
single_match=***true***

// The comparison is between experimental spectra and theoretical spectra
searchMode=***projection***

// Once a PSM is selected according to the number of shared peaks to interpret one spectrum, SpecFit tries to position ΔM on all sites in the peptide to generate different modified peptides in turn.
PSM are re-ranked according to the new number of shared peaks
shift=***true***
// Only the PSMs with at least 5 shared peaks are selected before SpecFit
threshold=**7**

```
// When PSMs are ranked to interpret a spectrum, a ΔM=0
interpretation is preferred if there is at least 8 shared
peaks.
minimumScore=60
// The lower bound of the precursor mass window
minMassDelta=-500
```

When the comparison was limited to the single file, no decoy database was used, the `minimumScore` *was set to 9,and threshold=6*

We obtain a large list of PSMs, from which we extract a subset of PSMs referring to peptide DATNVGDEGGFAPNIIENK. We run SpecGlobX on this subset with the default parameters (see supplementary datafile *config.parameters* in Additional File 1).

We obtained a list of 77 alignments. We extracted the related spectra to generate a small mgf file added as a demonstration file (see Additional File 1). This subset of spectra was also used with MODPlus and MSFragger.

C.1 MODPLus and MSFragger

Parameters were set as described above (section A), except the minimum/maximum modification size in Dalton that was enlarged when possible (Philosopher did not tolerate a minimum size below -230 Da)
ModMassSize=-500,3000

D. Annotated Spectrum Plots

Annotated spectra plots have been obtained using SeeMS.

- **Scan # 54484** interpreted as DATNVGDEGGFAPNILENK_[302,99]

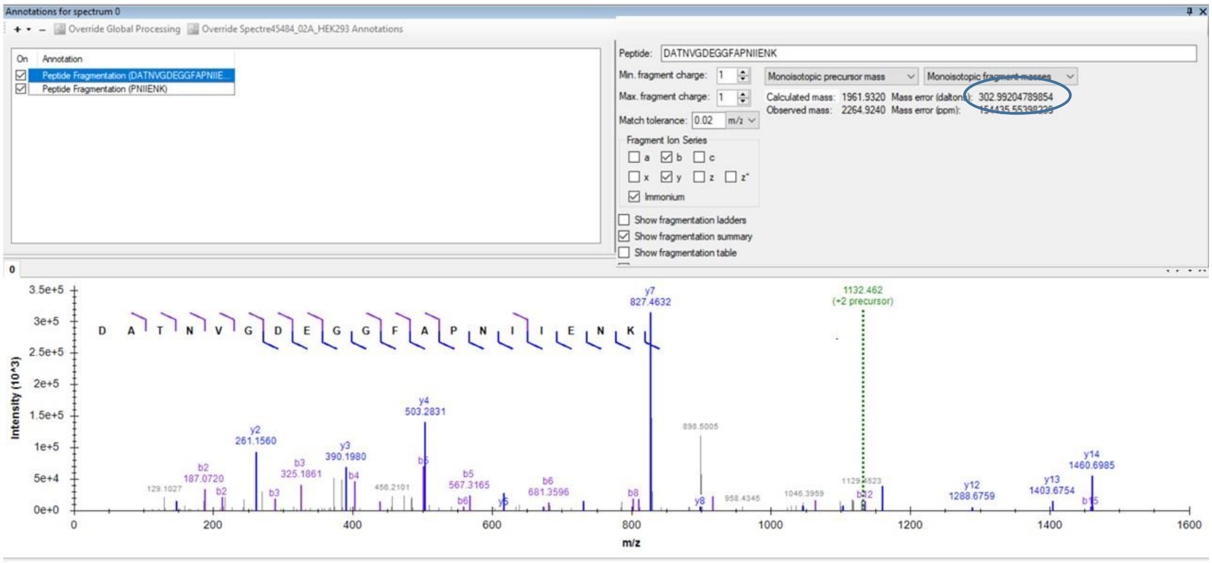

- **Scan # 54025** interpreted as YGKDATN[1914.72]VGDEGGFAPNILENK

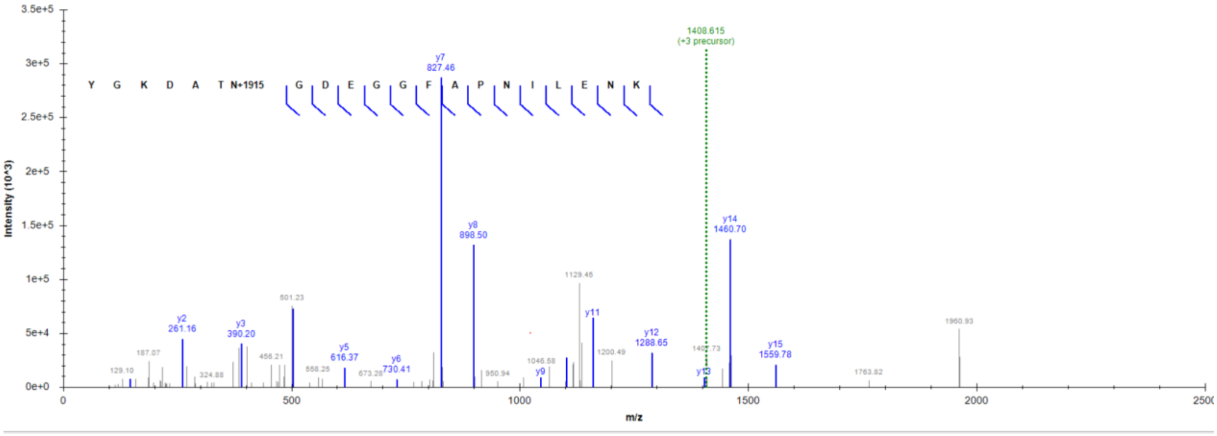

- **Scan # 54025** interpreted as a homodimer of DATNVGDEGGFAPNILENK] added to the frequent neutral loss 301.98 Da:

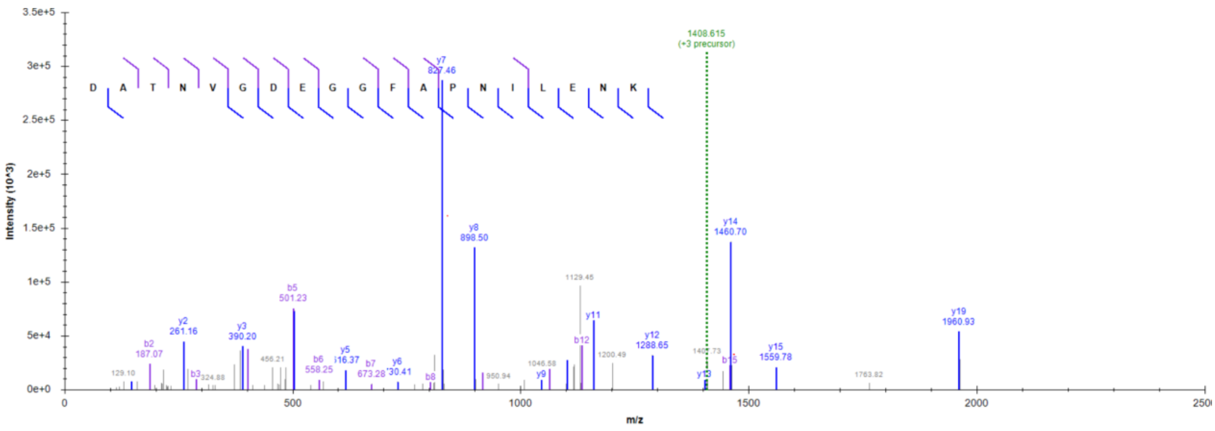

- **Scan # 53811** interpreted as peptide DATNVGDEGGFAPNILENK charged 3+

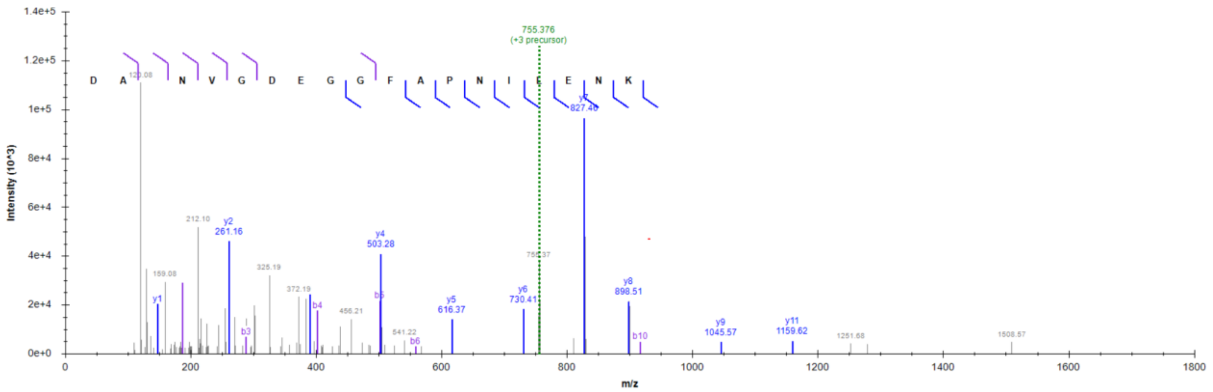

In the plot above, we can observe many intense peaks that are not annotated on the left of the graphic. Since the peptide contains a proline that frequently leads to internal fragmentation, we try to superpose peptide DATNVGDEGGFAPNILENK with peptide PNILENK, giving rise to a more convincing interpretation below:

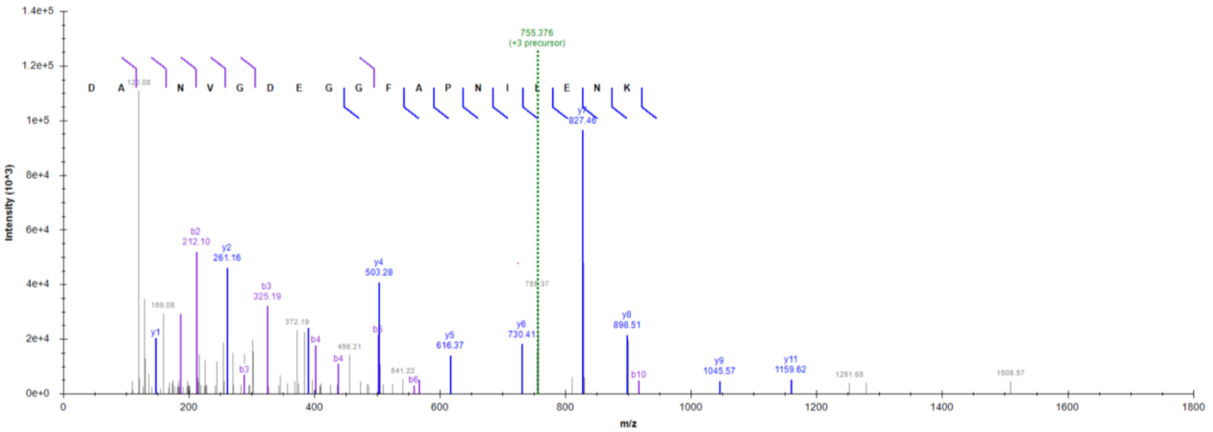

Supplement: Supplementary file 1 — Additional file 1. Complementary information concerning the evaluation of SpecGlobX. Test parameters and additional results on simulated spectra are detailed. Some spectral alignments are also presented. [file 12859_2023_5555_MOESM1_ESM.pdf]
